# Angiopoietin2 is associated with coagulation activation and tissue factor expression in extracellular vesicles in COVID-19

**DOI:** 10.3389/fmed.2024.1367544

**Published:** 2024-05-13

**Authors:** Mayck Silva Barbosa, Franciele de Lima, Carla Roberta Peachazepi Moraes, Ivanio Teixeira Borba-Junior, Stephany Cares Huber, Irene Santos, Bruna Bombassaro, Sergio San Juan Dertkigil, Anton Ilich, Nigel S. Key, Joyce M. Annichino-Bizzacchi, Fernanda Andrade Orsi, Eli Mansour, Licio A. Velloso, Erich Vinicius De Paula

**Affiliations:** ^1^School of Medical Sciences, Universidade Estadual de Campinas, Campinas, Brazil; ^2^Hematology and Hemotherapy Center, Universidade Estadual de Campinas, Campinas, Brazil; ^3^Obesity and Comorbidities Research Center, Universidade Estadual de Campinas, Campinas, Brazil; ^4^Blood Research Center, University of North Carolina, Chapel Hill, NC, United States; ^5^Division of Hematology, Department of Medicine, University of North Carolina, Chapel Hill, NC, United States; ^6^Department of Pathology and Laboratory Medicine, University of North Carolina, Chapel Hill, NC, United States

**Keywords:** COVID-19, hemostasis, inflammation, coagulation/thrombosis, angiopoietin, immunothrombosis

## Abstract

Coagulation activation in immunothrombosis involves various pathways distinct from classical hemostasis, offering potential therapeutic targets to control inflammation-induced hypercoagulability while potentially sparing hemostasis. The Angiopoietin/Tie2 pathway, previously linked to embryonic angiogenesis and sepsis-related endothelial barrier regulation, was recently associated with coagulation activation in sepsis and COVID-19. This study explores the connection between key mediators of the Angiopoietin/Tie2 pathway and coagulation activation. The study included COVID-19 patients with hypoxia and healthy controls. Blood samples were processed to obtain platelet-free plasma, and frozen until analysis. Extracellular vesicles (EVs) in plasma were characterized and quantified using flow cytometry, and their tissue factor (TF) procoagulant activity was measured using a kinetic chromogenic method. Several markers of hemostasis were assessed. Levels of ANGPT1, ANGPT2, and soluble Tie2 correlated with markers of coagulation and platelet activation. EVs from platelets and endothelial cells were increased in COVID-19 patients, and a significant increase in TF^+^ EVs derived from endothelial cells was observed. In addition, ANGPT2 levels were associated with TF expression and activity in EVs. In conclusion, we provide further evidence for the involvement of the Angiopoietin/Tie2 pathway in the coagulopathy of COVID-19 mediated in part by release of EVs as a potential source of TF activity.

## Introduction

Coagulation activation is an integral part of the host response to pathogens in a process termed “immunothrombosis” (1), best described in sepsis (2) and COVID-19 (3). Coagulation activation in immunothrombosis occurs through multiple pathways, including mechanisms that are not critical to classical, non-inflammatory hemostasis (4). Accordingly, knowledge about these mechanisms can reveal novel therapeutic targets to modulate the hypercoagulability observed in inflammatory conditions, with less impact on classical hemostasis.

The Angiopoietin/Tie2 pathway is one such mechanism, which until recently was only known to be associated with embryonic angiogenesis (5) and endothelial barrier regulation in sepsis (6). However, in 2018, this pathway was linked to coagulation activation in sepsis by a mechanism that involves tissue factor (TF) expression by endothelial cells (7). Specifically, activation of the tyrosine kinase receptor Tie2 by ANGPT1 has been shown to promote endothelial cell quiescence, while increased levels of ANGPT2 have been associated with endothelial and coagulation activation. Even more recently, the participation of this pathway in the pathophysiology of COVID-19 was demonstrated, suggesting its potential role as a therapeutic target (8).

EV are important elements of immunothrombosis since they carry TF and contribute to coagulation activation in several clinical contexts (9). EVs have been associated with hypercoagulability and disease severity in COVID-19 (10, 11).

In this study, we describe the association of key mediators of the *Angiopoietin*/Tie2 pathway with coagulation activation, TF expression and activity in EVs in a cohort of patients with COVID-19.

## Materials and methods

### Study population

This study was conducted in a previously described cohort (12), consisting of patients with COVID-19 who required hospital admission due to hypoxia, and were recruited to a clinical trial between April and June 2020 in an academic tertiary hospital. Only samples obtained before any trial intervention were used. Inclusion criteria were: age ≥ 18 years old, a positive RT-PCR for SARS-CoV-2, presence of typical COVID-19 pneumonia in lung CT scan, Sat O_2_ ≤ 94% in ambient air or PaO_2_ /FiO_2_ < 300 mmHg. Exclusion criteria included pregnancy, severe renal or liver disease, HIV infection or other immunodeficiency state, previous diagnosis of cancer, ischemic myocardial disease, history of thromboembolic events, or use of any experimental treatment for SARS-CoV-2 infection. Age and sex-matched healthy individuals from the same geographic region were recruited among healthcare employees of the local blood bank, observing the same exclusion criteria. The study was performed in accordance with the Declaration of Helsinki and approved by the Institutional Review Board.

### Sample collection and processing

Blood samples were collected into 3.2% sodium citrate or EDTA-K2 tubes within 24 h from the diagnosis of COVID-19 and processed within 2 h of collection by double centrifugation at 1,800 g for 15 min at 22° C to obtain platelet-free plasma. EDTA-K2 samples were only submitted to the first cycle of centrifugation. Plasma aliquots were immediately frozen at −80°C until analysis.

### Clinical and laboratory outcomes

Clinical and laboratory data were obtained from electronic medical records and from case report forms of the clinical trial and have been previously reported in more detail (11).

### Characterization and enumeration of EVs

To characterize and quantify EVs in plasma samples, flow cytometry was performed as previously described (13). Platelet-free plasma was thawed at 37°C for 3 min and incubated on ice for 30 min to identify cell derived-EV from platelets, endothelial cells or erythrocytes. To platelet-free plasma a reagent mix was added that contained Calcein Violet (AM, Thermo Fisher Scientific) for cell membrane integrity, Bovine Lactadherin (FITC, Haematologic Tech) for EV identification, anti-CD41 (APC, eBioscience™) for platelets, anti-CD146 (eFluor™ 660, eBioscience™) for endothelial cells, anti-CD235a (APC, eBioscience™) for erythocytes, anti-CD142 (PE, eBioscience™) for TF and filtered sterile phosphate-buffered saline (PBS) buffer (Hyclone without calcium, magnesium and phenol red). Cell-derived EVs were identified as events that were positive for lactadherin, calcein and the respective antibody (CD235a, CD41 or CD146). After incubation, samples were centrifuged at 20,000 *g* for 30 min at 4°C. EV pellets were then resuspended in a 1.0 mL of PBS buffer and analyzed in a CytoFLEX cytometer (Beckman Coulter, Carlsbad, CA, USA). Rosetta beads (Exometry, Amsterdam, the Netherlands) were used for calibration. For standardization, FMO control (Fluorescence Minus One) was performed. PBS buffer was used as a negative control between each sample, and EVs were expressed as events per microliter.

### EV-tissue factor procoagulant activity

Measurement of tissue factor-dependent procoagulant activity (TF-PCA) was performed in EVs extracted from citrated plasma. A kinetic chromogenic method was used based on the generation of factor Xa (14). Of note, these results have been previously reported elsewhere (11).

### Other markers of coagulation activation

D-dimer was measured using an immunoturbidimetric assay (Innovance D-dimer, Siemens Healthcare). ANGPT1, ANGPT2, P-selectin, urokinase-type plasminogen activator receptor (PLAUR) and plasminogen activator inhibitor 1 (SERPINE1) levels were measured using a customized Luminex immunoassay (Procarta Plex multiplex panel, Thermo-Fischer Scientific) in a Bioplex 200 instrument (Bio-Rad). Thrombin-antithrombin complex (TAT; Molecular Innovations), soluble Tie2 (R&D Systems) and plasmin-antiplasmin complex (PAP; Technozym®) levels were measured using commercially available ELISA kits. All assays were performed in citrate-anticoagulated plasma, except for the Luminex immunoassay, which was performed in EDTA-anticoagulated plasma.

### Statistical analysis

Data are presented as mean ± standard deviation (SD) or as medians and interquartile range (IQR), as indicated. Differences in continuous variables were compared using the Student’s t-test or Mann–Whitney test, according to data distribution, assessed by the D’Agostino & Pearson normality test. Correlation was calculated using the Pearson or Spearman correlation coefficient. A *p* value ≤0.05 was considered significant. Receiver operating characteristics (ROC) curves were generated with a regression logistic model that included the EV TF-PCA to assess the predictability of an ANGPT2 level above 5,000 pg/mL. All statistical analyses were performed using SPSS version 26 (IBM) or GraphPad Prism 8.0 Software (GraphPad Inc).

## Results

### Patient characteristics

The study population consisted of 30 consecutive patients with COVID-19 and 30 healthy individuals. The main clinical and laboratory characteristics of the study population are shown in Table 1. As expected, several markers of inflammation, coagulation and fibrinolysis activation were increased in patients compared to healthy controls. Proteins of the Angiopoietin/Tie2 pathway were also increased in patients, as previously described (15).

**Table 1:** Clinical and laboratory characteristics of the study population.

	Patients (*n* = 30)	Healthy individuals (*n* = 30)	*p*-value^‡^
Age^†^	52.7 ± 12.3	50.3 ± 9.2	0.40
Sex, male:female	16:14	16:14	1.00
Body mass index^†^	30.6 ± 6.6	25.9 ± 4.2	0.006
Neutrophils, *10^9^/L^†^	6.38 ± 3.77	3.09 ± 0.93	< 0.001
Lymphocytes, *10^9^/L^†^	1.20 ± 0.55	1.79 ± 0.28	<0.001
Platelets, *10^9^/L^†^	216.33 ± 93.02	245.59 ± 40.34	0.12
**Clinical outcomes**			
Time from symptom onset, days^†^	8.1 ± 2.3	-	-
Need of O_2_ support, days ^¶^ %^†^	11.4 ± 9.3	-	-
Extent of lung disease (CT score)^†^	17.8 ± 7.3	-	-
Length of hospital stay, days^†^	13.4 ± 9.6	-	-
Need for intensive care (%)^†^	12/30 (40%)	-	-
Length of intensive care stay, days^†^	6.1 ± 9.7	-	-
**Laboratory markers of hemostasis activation**			
C-reactive protein, mg/L*	96.3 (53.2–157.0)	2.0 (1.0–4.0)	<0.0001
D-dimer, ng/mL*	760 (625–1,197)	243.0 (150–509)	<0.0001
TAT, ng/mL*	2.85 (2.23–3.59)	2.25 (1.83–2.63)	0.008
F8, U/dL^†^	212.9 ± 92.0	132.1 ± 31.3	<0.0001
VWFAg, U/dL*	248 (238–458)	145 (102–208)	<0.0001
Antithrombin, %^†^	114.1 ± 17.9	109.4 ± 10.5	0.26
SELPLG, pg/mL^†^	3,540.5 ± 1,773.8	2,926 ± 1,352.7	0.14
PLAUR, ng/mL^†^	1.80 ± 0.57	1.08 ± 0.30	<0.001
*SERPINE1*, pg/mL^†^	702.1 ± 153.3	588.4 ± 194.7	0.015
PAP, mg/mL*	1.24 (0.99–2.10)	0.35 (0.30–0.46)	<0.001
EV TF-PCA, pg/mL*	0.04 (0.01–0.21)	0.01 (0.00–0.03)	0.003
**Ang/Tie2 pathway mediators**			
ANGPT1 pg/mL^†^	463.2 ± 194.6	237.4 ± 104.9	<0.0001
ANGPT2 pg/mL*	1,926 (1,275 – 3,134)	1,215 (927.0–1,445)	<0.0001
soluble Tie2 pg/mL^†^	10,753 ± 2.377	8,603 ± 1.851	<0.0001

^†^mean ± SD; *Median (interquartile range); ^‡^Mann–Whitney test or *t*-test for Gaussian or non-Gaussian data, respectively. TAT, thrombin-antithrombin complex; F8, factor VIII; VWFAg, Von Willebrand Factor antigen; PLAUR, urokinase-type plasminogen activator receptor; SERPINE1, plasminogen activator inhibitor-1; PAP, plasmin-antiplasmin complex; EV TF-PCA, extracellular vesicle tissue factor procoagulant activity. ANGPT1, angiopoietin1; ANGPT2, angiopoietin2. SELPLG, selectin P ligand.

### Mediators of the ANGPT/Tie2 pathway are associated with coagulation and fibrinolysis activation

**Figure 1:**
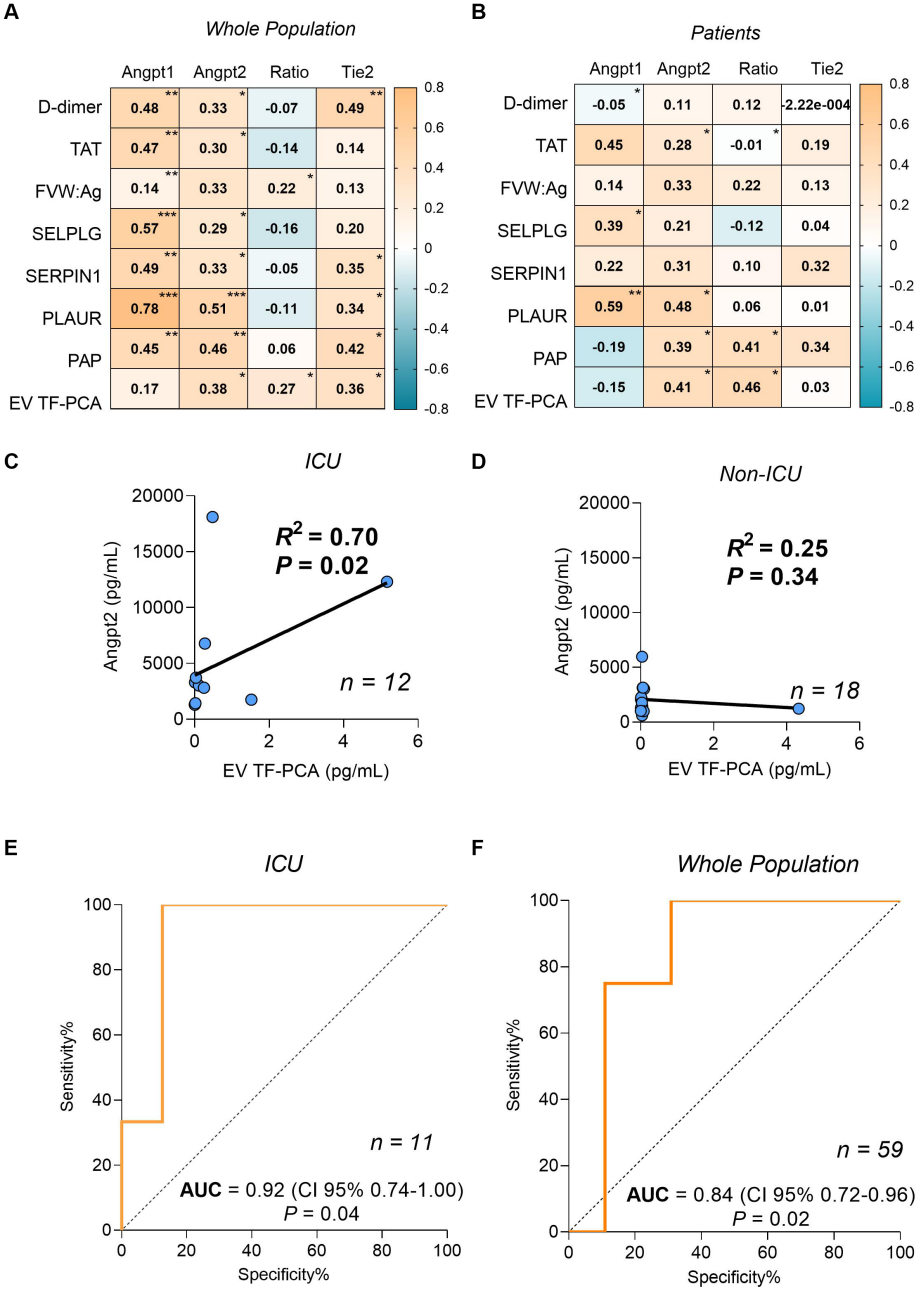
Spearman correlation coefficients matrix between ANGPT1, ANGPT2, ANGPT2/ANGPT1 ratio, Tie2 and coagulation/fibrinolysis markers from the whole population **(A)** and only patients **(B)**. Correlation between plasma levels of ANGPT2 and EV TF-PCA in patients who required intensive care unit (ICU) admission **(C)**, and non-ICU patients **(D)**. ROC analysis for the prediction of ANGPT2 levels higher than 5,000 pg/mL by EV TF-PCA measurement of ICU patients (*n* = 11) **(E)** or ICU and the whole population (*n* = 59) **(F)**. (*p* < 0.0001)***; (≥ 0.0001 *p* ≤ 0.001)**; (>0.001 *p* ≤ 0.05)*. TAT, thrombin-antithrombin complex; FVIII, factor VIII activity; VWF, Ag: Von Willebrand Factor; PLAUR, urokinase-type plasminogen activator receptor; SERPINE1: plasminogen activator inhibitor-1; PAP, plasmin-antiplasmin complex; EV TF-PCA, extracellular vesicle tissue factor procoagulant activity.

ANGPT1, ANGPT2 and Tie2 levels were consistently correlated with markers of coagulation and fibrinolysis activation (Figures 1A,B). Since ANGPT2 levels have been consistently associated with disease severity in sepsis and COVID-19 (15), we further explored this association in our study population. Interestingly, a strong correlation between ANGPT2 levels and EV TF-PCA was observed in patients who required ICU admission (*r*^2^ = 0.70, *p* = 0.02, *n* = 12) (Figure 1C), but not in non-ICU patients (*r*^2^ = 0.25, *p* = 0.34, *n* = 18) (Figure 1D). The association between circulating ANGPT2 levels and EV TF-PCA was further confirmed by ROC analysis demonstrating that an ANGPT2 level above 5,000 pg/mL, which has been previously associated with COVID-19 severity (16), could be predicted with an estimated accuracy of 0.92 (CI95% 0.74–1.00; *p* = 0.04) and 0.84 (CI95% 0.72–0.96; *p* = 0.02) by EV TF-PCA levels in the subset of patients who required ICU management (Figure 1E), and in the whole study population (patients and healthy individuals) (Figure 1F).

### Higher ANGPT2 levels are associated with increased counts of TF-expressing EVs from endothelial cells

**Figure 2:**
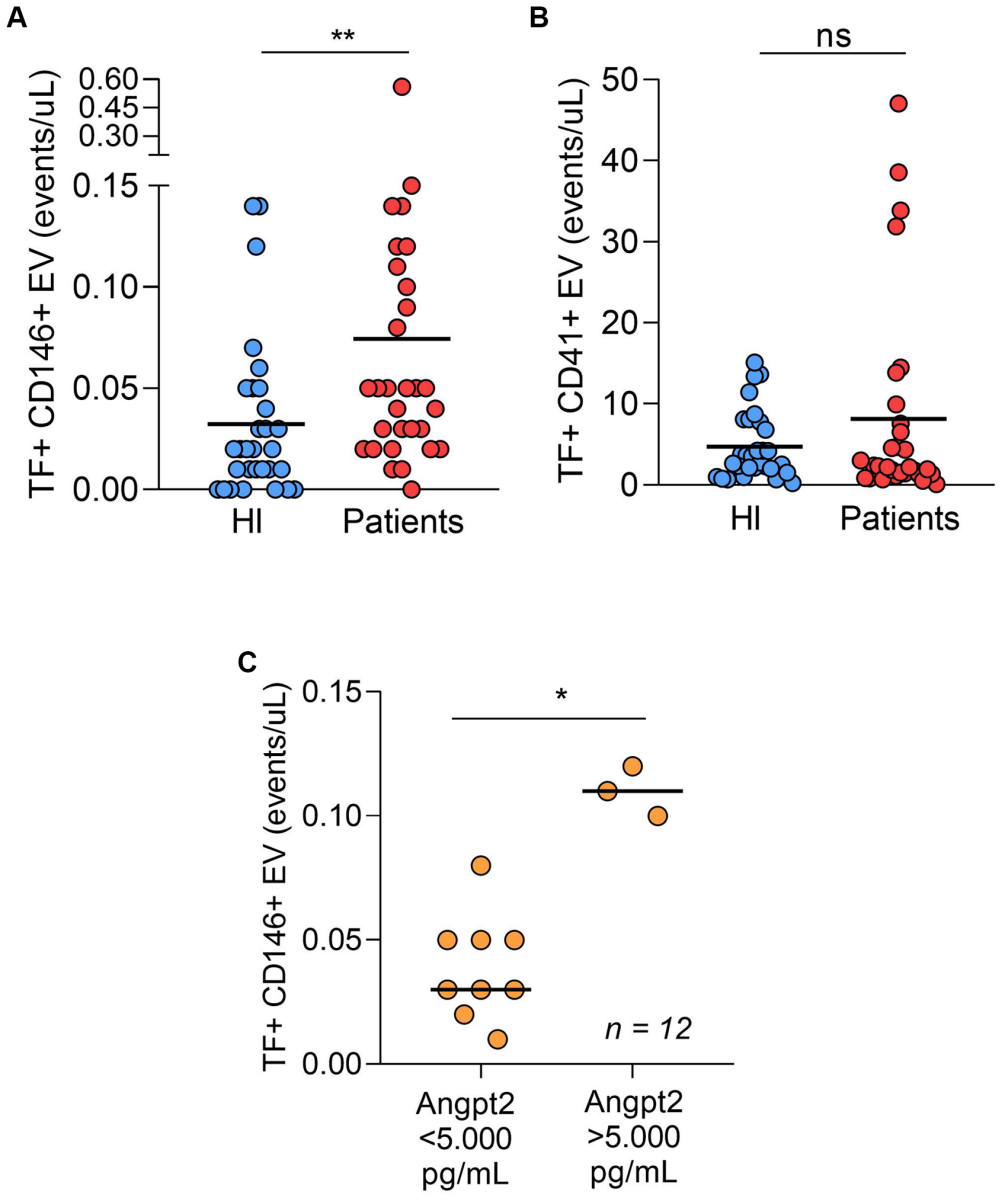
TF-positive endothelial cell derived-EV **(A)** and TF-positive platelet derived-EV **(B)** in patients and healthy individuals (*n* = 30 per group). In ICU patients (*n* = 12), both ANGPT2 levels were higher in patients with higher counts of TF-expressing EV **(C)**. The cut-off of 5,000 pg/mL was based on a previous study in COVID-19. (*p* = 0.003)**; (*p* = 0.004)*; ns, non-significant. Bar indicates median value. HI, healthy individuals.

In our study population, both platelet- and endothelial cell-derived EV were more frequent in patients compared to controls (Table 2). We and others have demonstrated that EV-mediated TF-PCA is increased in COVID-19 (10, 11, 17). Interestingly, when TF expression was assessed in these EVs, only endothelial cell-derived EV presented higher TF expression (Figures 2A,B and Supplementary Table S3). Moreover, in patients who required ICU, ANGPT2 levels segregated with higher TF expression in endothelial cell-derived EV (Figure 2C), suggesting an association between ANGPT2 and TF expression from endothelial cells, as previously described in sepsis (7).

**Table 2:** Total extracellular vesicles count in COVID-19 patients at admission.

	Patients (*n* = 30)	Healthy individuals (*n* = 30)	*p*-value^‡^
Platelets (CD41^+^), events/ μL^¶^	137.2 ± 74.5	37.0 ± 17.0	<0.0001
EC (CD146^+^), events/ μL*	0.16 (0.09–0.25)	0.05 (0.02–0.08)	<0.0001
RBC (CD235^+^), events/ μL*	0.4 (0.02–1.1)	0.3 (0.1–0.7)	0.4
Leukocytes (CD45^+^), events/ μL*	2.15 (1.08–2.82)	1.36 (0.80–2.82)	0.2
Monocytes (CD14^+^), events/ μL*	0.47 (0.23–0.95)	0.56 (0.28–0.89)	0.9

*Median (interquartile range); ^¶^mean ± SD; ^‡^Mann–Whitney test or *t*-test for Gaussian or non-Gaussian distributed data, respectively. EC, endothelial cells; RBC, red blood cells.

## Discussion

Considering the heterogeneity of mechanisms of coagulation activation in inflammatory conditions, our study aimed to explore whether Angiopoietin/Tie2 pathway mediators, which are well-known biomarkers of severity in COVID-19, are associated with hypercoagulability. Our main results were the demonstration that ANGPT1, ANGPT2 and soluble Tie2 levels correlate with several markers of coagulation and platelet activation, and that ANGPT2 levels are associated with TF expression and activity in EVs obtained from these patients.

The crosstalk between Angiopoietin/Tie2 pathway and coagulation activation was initially demonstrated in sepsis (7). Using animal models, it was demonstrated that phosphorylation of the tyrosine kinase Tie2 in endothelial cells by ANGPT1 maintained these cells in a quiescent state, which could be reversed by the downregulation of Tie2 phosphorylation by the competitive ligand ANGPT2. Moreover, Tie2 activation by ANGPT1 dowregulated the expression of TF (7). The same group demonstrated that plasma from patients with COVID-19 was capable of inducing gene expression of several mediators of immunothrombosis, including *ANGPT2* and *F3*, in cultured endothelial cells (8). More recently, a new mechanism linking ANGPT2 to coagulation activation was proposed, involving the binding of ANGPT2 to thrombomodulin, thus inhibiting its anticoagulant effect (18). The clinical association between ANGPT2 levels and disease severity in COVID-19 has been extensively demonstrated (15, 16, 19–21). We confirmed and extended these findings, first by demonstrating in human samples that biomarkers encompassing several elements of hemostasis correlate with ANGPT1 and ANGPT2. While the magnitude of these correlations was mostly mild to moderate, with the exception of soluble PLAUR, the consistency of the statistical association reinforces the concept that these mechanisms are linked. Of note, EV TF-PCA and PAP, which are well-known markers of hemostasis and/or fibrinolysis activation, were correlated the ANGPT2/ANGPT1 ratio. The stronger correlation coefficients were those between Angiopoietin/Tie2 and fibrinolytic pathways, namely soluble PLAUR, but also SERPINE1. The importance of fibrinolysis in the pathogenesis of COVID-19 is suggested by the striking association of D-dimer with disease severity (22, 23) and by the fact that SERPINE1 has also been associated with prognosis in these patients (24). The prominence of intra-alveolar fibrin accumulation in the pathogenesis of COVID-19 could explain why the magnitude of fibrinolytic activation is an important element in the pathogenesis of this condition, and our data demonstrate an association of this process with imbalances in the Angiopoietin/Tie2 pathway activation. Of note, soluble PLAUR is also a biomarker of disease severity in other conditions associated with immunothrombosis and alveolar damage such as sepsis (25, 26). Another pathway that could link increased PLAUR levels with coagulation activation is the association of this molecule with factor XII and neutrophil activation, which has been demonstrated in wound healing (27) and sickle cell disease (28).

One intriguing finding was that ANGPT1, which one would expect to behave in the opposite direction of ANGPT2 in regard to the correlation with markers of coagulation activation, was also positively associated with several markers of hemostasis activation. We speculate the immunothrombotic response observed in COVID-19 leads to the release of both mediators. It should be noted, however, that when only patients are included in the analysis, the absolute value of the correlation coefficient between ANGPT2 with EV TF-PCA is positive for ANGPT2 and negative for ANGPT2, yielding a statistically significant positive correlation between EV TF PCA and the ANGPT2/ANGPT1 ratio, in accordance with our working hypothesis.

The release of EVs has been associated with several inflammatory conditions (29–31), including COVID-19 (17, 32). Here we confirmed that EVs from platelets and endothelial cells are increased in COVID-19, and that endothelial cells, but not platelets, represent a significant source of TF+ EVs in these patients. These results are aligned with previous findings showing that EVs from endothelial cells were increased in COVID-19 patients who presented with thromboembolic events (17). It has previously been shown that ANGPT2 levels above 5,000 pg/mL could identify patient with a worse prognosis among COVID-19 ICU patients (16). Interestingly, in our cohort, patients with ANGPT2 levels higher than 5,000 pg/mL at admission showed both higher counts of TF+ EVs from endothelial cells, as well as higher TF procoagulant activity, reinforcing the concept that ANGPT2 could be associated with TF expression in COVID-19. At least two mechanisms could explain this association. Constitutive Tie2 signaling would maintain endothelial quiescence, while increase in ANGPT2 levels observed in inflammatory situations would temporarily disturb Tie2 activation (5). This could lead to endothelial barrier disruption, which has also been associated with ANGPT2 levels in COVID-19 (15), which in turn could promote the release of EVs from endothelial cells (Supplementary Figure S3). Another mechanism by which ANGPT2 could contribute to the prothrombotic state observed in COVID-19 is by thrombomodulin-dependent inhibition of the protein C pathway, as recently postulated (18).

This study has limitations that need to be acknowledged. First, it represents an exploration of the association between two pathways (Angiopoietin/Tie2 and hemostasis) which have been previously shown to interact, but it does not include experiments that allow us to definitely confirm this crosstalk in the context of COVID-19. In this regard, our results add incremental evidence to the concept that activation of the Angiopoietin/Tie2 pathway contributes to coagulation activation in COVID-19, paving the way for mechanistic and/or interventional studies in this area. Second, although patients with PE or DVT would represent a very interesting group to address this question, the COVID-19 patient cohort served as the basis for our study, and it was decided to use them to address this question because higher ANGPT2 levels in this population are linked to the severity of the disease (and may therefore be involved in its pathogenesis) and also show clinical and laboratory indicators of hypercoagulability.

Third, the number of patients is relatively small, so that the study may have been underpowered to demonstrate some associations. However, the characteristics of the cohort, derived from a randomized clinical trial, allowed us to work with a well-defined population both in terms of the clinical characteristics, and in the quality of clinical and outcome data, as well as uniform biological samples. Finally, the accuracy of methods that measure TF protein in EVs has been recently debated (33), based on the assumption that functional assays are a more biologically relevant method to measure TF expression. In our TF protein assays, FMO controls were used during the standardization phase, but isotype controls were not, so that our results should be interpreted with caution. However, the association between ANGPT2 levels and TF expression was confirmed using a well-established activity assay (34).

In conclusion, the Angiopoietin/Tie2 pathway is disturbed in COVID-19 and associated with coagulation and fibrinolysis activation, and with increased release of TF+ EVs from endothelial cells on admission. Additional studies regarding the effect of Angiopoietin/Tie2 mediators on TF expression in EVs are warranted to confirm our findings, and to identify additional therapeutic targets that regulate immunothrombosis in COVID-19 and other viral respiratory diseases.

## Data availability statement

The original contributions presented in the study are included in the article/Supplementary material, further inquiries can be directed to the corresponding author.

## Ethics statement

The studies involving humans were approved by Research Ethics Committee of the Universidade Estadual de Campinas (protocol number CAAE 36528420.3.0000.5404). The studies were conducted in accordance with the local legislation and institutional requirements. The participants provided their written informed consent to participate in this study.

## Author contributions

MB: Formal analysis, Investigation, Methodology, Writing – original draft, Writing – review & editing. FL: Investigation, Methodology, Writing – review & editing. CP: Investigation, Methodology, Writing – review & editing. IB-J: Investigation, Methodology, Writing – review & editing. SH: Investigation, Methodology, Writing – review & editing. IS: Investigation, Methodology, Writing – review & editing. BB: Investigation, Methodology, Writing – review & editing. SD: Investigation, Methodology, Writing – review & editing. AI: Investigation, Methodology, Writing – review & editing. NK: Investigation, Supervision, Writing – review & editing. JA-B: Investigation, Writing – review & editing. FO: Investigation, Writing – review & editing. EM: Investigation, Methodology, Writing – review & editing. LV: Conceptualization, Investigation, Methodology, Writing – review & editing. EP: Conceptualization, Data curation, Formal analysis, Investigation, Methodology, Project administration, Resources, Software, Supervision, Validation, Visualization, Writing – original draft, Writing – review & editing.
