# Supplementary material for: Angiopoietin2 is associated with coagulation activation and tissue factor expression in extracellular vesicles in COVID-19
Source: Front Med (Lausanne). 2024 May 13;11:1367544. doi: 10.3389/fmed.2024.1367544 (PMC11128612; doi:10.3389/fmed.2024.1367544)
Supplement: Supplementary file 1 [file Table_3.DOCX]

Supplementary Material

**Angiopoietin2 is associated with coagulation activation and tissue factor expression in extracellular vesicles in COVID-19**

[Mayck Silva Barbosa](https://pubmed.ncbi.nlm.nih.gov/?term=Barbosa+MS&cauthor_id=35235941), [Franciele de Lima](https://pubmed.ncbi.nlm.nih.gov/?term=Lima+F&cauthor_id=35235941), [Carla Roberta Peachazepi Moraes](https://pubmed.ncbi.nlm.nih.gov/?term=Moraes+CRP&cauthor_id=35235941), [Ivanio](https://pubmed.ncbi.nlm.nih.gov/?term=Moraes+CRP&cauthor_id=35235941) Teixeira Borba-Junior, [Stephany Cares Huber](https://pubmed.ncbi.nlm.nih.gov/?term=Huber+SC&cauthor_id=35235941), [Irene Santos](https://pubmed.ncbi.nlm.nih.gov/?term=Santos+I&cauthor_id=35235941), [Bruna Bombassaro](https://pubmed.ncbi.nlm.nih.gov/?term=Bombassaro+B&cauthor_id=35235941), [Sergio San Juan Dertkigil](https://pubmed.ncbi.nlm.nih.gov/?term=Dertkigil+SSJ&cauthor_id=35235941),  [Anton Ilich](https://pubmed.ncbi.nlm.nih.gov/?term=Ilich+A&cauthor_id=35235941), [Nigel S Key](https://pubmed.ncbi.nlm.nih.gov/?term=Key+NS&cauthor_id=35235941), [Joyce M Annichino-Bizzacchi](https://pubmed.ncbi.nlm.nih.gov/?term=Annichino-Bizzacchi+JM&cauthor_id=35235941) , [Fernanda Andrade Orsi](https://pubmed.ncbi.nlm.nih.gov/?term=Orsi+FA&cauthor_id=35235941), [Eli Mansour](https://pubmed.ncbi.nlm.nih.gov/?term=Mansour+E&cauthor_id=35235941), [Licio A Velloso](https://pubmed.ncbi.nlm.nih.gov/?term=Velloso+LA&cauthor_id=35235941), [Erich Vinicius De Paula](https://pubmed.ncbi.nlm.nih.gov/?term=De+Paula+EV&cauthor_id=35235941)*****

*****Corresponding Author: erich@unicamp.br

# Supplementary Table 3. TF-positive extracellular vesicles count in COVID-19 patients at admission

|  | Patients  (n=30) | Healthy individuals  (n=30) | *P* value^‡^ |
| --- | --- | --- | --- |
| Platelets (CD41^+^ TF^+^), events/ µL* | 2.2 (1.36 – 8.16) | 3.2 (1.89 – 7.84) | 0.8 |
| EC (CD146^+^ TF^+^), events/ µL* | 0.05 (0.02 – 0.10) | 0.02 (0.00 – 0.05) | 0.003 |
| RBC (CD235^+^ TF^+^), events/ µL * | 0.0 (0.00 – 0.01) | 0.0 (0.00 – 0.01) | 0.9 |
| Leukocytes (CD45^+^ TF^+^), events/ µL * | 1.8 (1.02 – 2.39) | 1.2 (0.52 – 2.56) | 0.3 |
| Monocytes (CD14^+^ TF^+^), events/ µL * | 0.1 (0.04 – 0.36) | 0.2 (0.05 – 0.35) | 0.5 |

*Median (interquartile range); ^¶^mean ± SD; ^‡^ Mann-Whitney test or t-test for Gaussian or non-Gaussian distributed data respectively. EC: endothelial cells; RBC: red blood cells.

**Supplementary** **Figure 3.** Increased Angpt2 levels is associated with TF-dependent coagulation activation on COVID-19 (Working hypothesis).

#
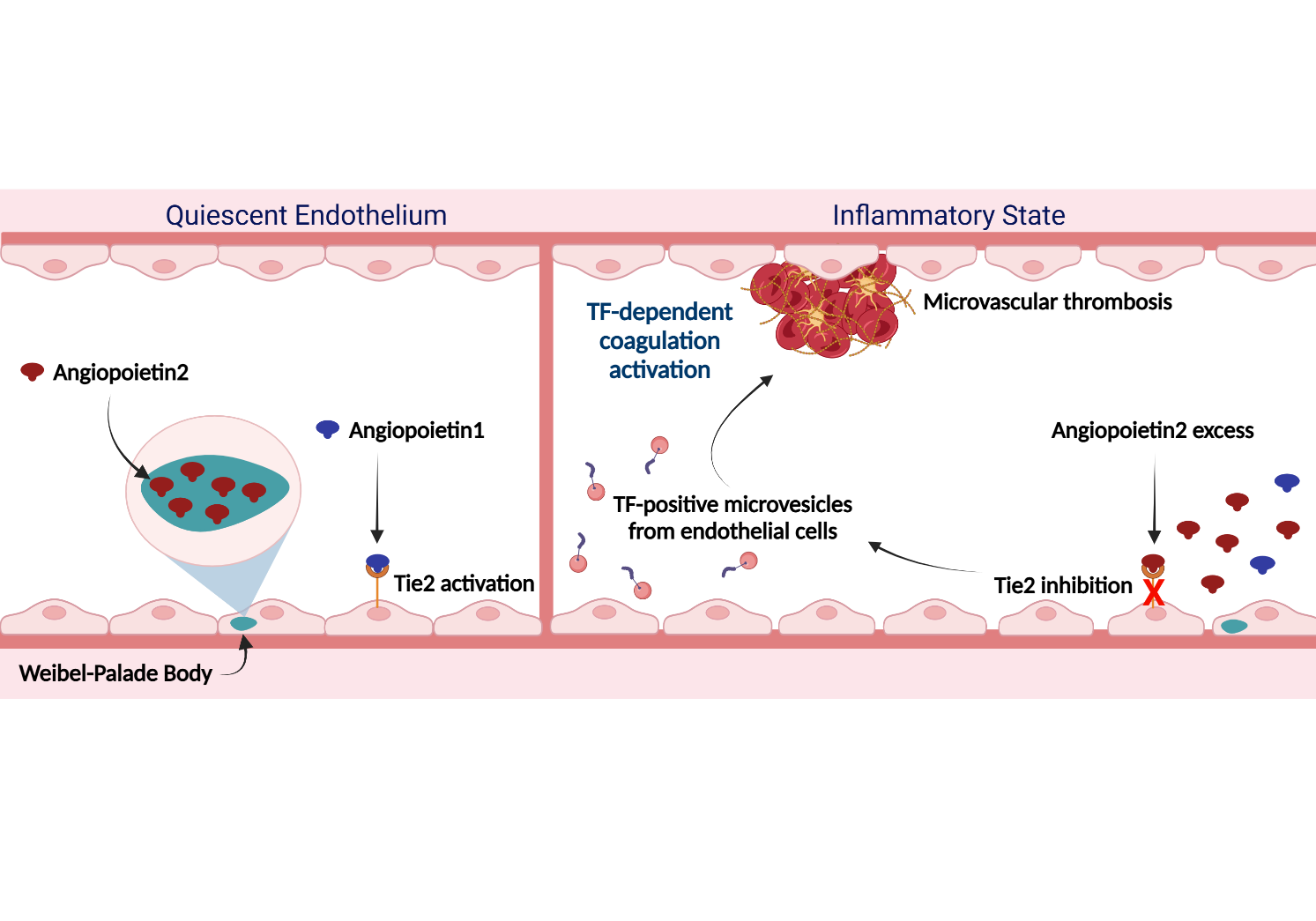


Created with BioRender.com
